# The Personal Genome Project-UK, an open access resource of human multi-omics data

**DOI:** 10.1038/s41597-019-0205-4

**Published:** 2019-10-31

**Authors:** Olga Chervova, Lucia Conde, José Afonso Guerra-Assunção, Ismail Moghul, Amy P. Webster, Alison Berner, Elizabeth Larose Cadieux, Yuan Tian, Vitaly Voloshin, Tiago F. Jesus, Rifat Hamoudi, Javier Herrero, Stephan Beck

**Affiliations:** 1 UCL Cancer Institute, University College London, London, UK; 2 Barts Cancer Institute, Queen Mary University of London, London, UK; 3 The Francis Crick Institute, London, UK; 4 School of Engineering, University of Warwick, Coventry, UK; 5 Lifebit Biotech Ltd., 219 Kensington High Street, London, W86BD UK; 6 College of Medicine, University of Sharjah, Sharjah, UAE; 7 Division of Surgery and Interventional Science, University College London, London, UK

**Keywords:** Personalized medicine, Data processing, Transcriptomics, Epigenomics, Quality control

## Abstract

Integrative analysis of multi-omics data is a powerful approach for gaining functional insights into biological and medical processes. Conducting these multifaceted analyses on human samples is often complicated by the fact that the raw sequencing output is rarely available under open access. The Personal Genome Project UK (PGP-UK) is one of few resources that recruits its participants under open consent and makes the resulting multi-omics data freely and openly available. As part of this resource, we describe the PGP-UK multi-omics reference panel consisting of ten genomic, methylomic and transcriptomic data. Specifically, we outline the data processing, quality control and validation procedures which were implemented to ensure data integrity and exclude sample mix-ups. In addition, we provide a REST API to facilitate the download of the entire PGP-UK dataset. The data are also available from two cloud-based environments, providing platforms for free integrated analysis. In conclusion, the genotype-validated PGP-UK multi-omics human reference panel described here provides a valuable new open access resource for integrated analyses in support of personal and medical genomics.

## Abstract (summary)

**Table:** 

Measurement(s)	DNA methylation profiling data • whole genome sequencing assay • bisulfite sequencing assay • transcription profiling assay
Technology Type(s)	DNA methylation profiling assay • DNA sequencing • RNA sequencing
Factor Type(s)	age • sex • smoking status
Sample Characteristic - Organism	Homo sapiens

Machine-accessible metadata file describing the reported data: 10.6084/m9.figshare.9896405

## Background & Summary

The Personal Genome Project UK (PGP-UK) is a member of the global PGP network together with the PGPs in the United States, Canada, Austria and China. The PGP network aims to provide multi-omics and trait data under open access to the community. This contributes to personalised medicine by advancing our understanding of how phenotypes and the development of diseases are influenced by genetic, epigenetic, environmental and lifestyle factors. While all five PGP centres generate whole-genome sequencing (WGS), some PGPs, such as PGP-UK, produce additional multi-omics data.

To participate in this study, volunteers must pass the eligibility criteria (e.g. be a UK citizen or permanent resident), sign the open consent form and pass a very thorough entrance exam. The objective of the exam is to ensure that the participant understands the key PGP-UK procedures and the potential risks of being involved in a project of this nature. At present, 1100 subjects have successfully enrolled in the project, and over a hundred of them have had their genomes sequenced. Once enrolled, participants are invited for sample collection which involves giving a blood or saliva sample or both for DNA and RNA extraction. DNA sequencing is then performed followed by data analysis. The results are reported back to the participants in the form of a Genome Report that is made publicly available after a grace period of one month. However, the participant is able to withdraw from the project at any time. DNA methylation data is generated using the Illumina HumanMethylation450 BeadChip array (450 k) and results are displayed in a freely available Methylome Report, a unique feature of the UK branch of the project. The preparation of both Genome and Methylome reports is discussed in more details in the Usage Notes Section.

**Fig. 1:**
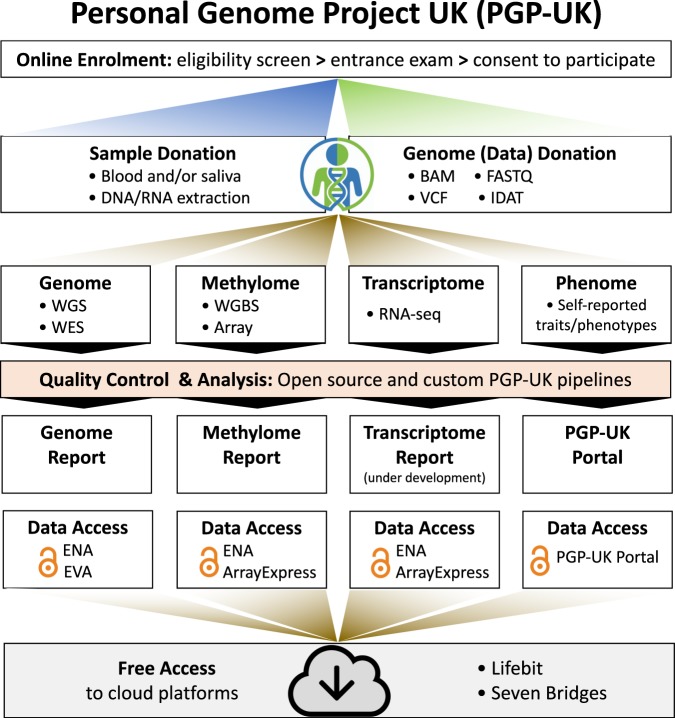
PGP-UK workflow. Horizontal panels depict the general sample/data categories and options (e.g blood and/or saliva) and vertical panels depict specific data types and their flow from start to end.

A pilot cohort of ten members of the public make up the PGP-UK multi-omics reference panel. For this cohort, we collected whole-genome bisulfite sequencing (WGBS) and RNA sequencing (RNA-seq) in addition to WGS and 450 k data. Figure 1 shows a schematic of the PGP-UK workflow. More information about PGP-UK can be found in^1,2^ and on the project’s website www.personalgenomes.org.uk.

While controlled access multi-omics data can be submitted into a single public repository (e.g. EGA in Europe or dbGaP in the USA), there is currently no single public repository for open access multi-omics data. Consequently, the different types of datasets (WGS, WGBS, RNA-seq, 450 k) were submitted to the corresponding repositories (European Nucleotide Archive (ENA), European Variation Archive (EVA), ArrayExpress) at EMBL-EBI. The details are given in the Data Records section and direct data download links are provided on the PGP-UK data web page www.personalgenomes.org.uk/data. For convenience, we offer a web API to download all the available PGP-UK data (see Data Records). The cumulative size of the PGP-UK multi-omics reference panel exceeds 2TB, which means that it would take over 3 days (more than 85 hours) to download (with mean UK download speed of 54.2 Mbps, Ofcom 2018). To overcome this limitation, we collaborated with two cloud platform providers (Seven Bridges Genomics and Lifebit) to host PGP-UK data in their respective clouds for unrestricted access as briefly described in Data Records section.

In this paper, we describe the PGP-UK multi-omics human reference panel derived from 10 participants. We followed best practices to perform various quality control (QC) checks to ensure the quality of the pilot WGS, WGBS, RNA-seq and 450 k datasets as described in the Technical Validation section. Finally, we describe the methods employed for multi-omics data matching, which ensures that samples are mapped to the correct participant.

## Methods

### Ethics

The PGP-UK study is approved by the University College London (UCL) Research Ethics Committee (ID Number 4700/001) subject to annual reviews and renewals. All the research activities in the project are conducted in accordance with the Declaration of Helsinki, UK national laws and medical research regulatory requirements. Prior to their enrolment, every participant must pass an entrance exam, give their consent to participate in the project and agree for their data and associated reports to be made publicly available under open access.

### Tissue samples

Blood samples were collected using EDTA Vacutainers (Becton Dickinson). Saliva samples were collected using Oragene OG-500 self-sampling kits. Sample processing and storage protocols were in line with HTA-approved standard operating procedures.

### Whole-genome sequencing (WGS)

WGS libraries were prepared from whole blood DNA using Illumina TruSeq Nano in accordance with standard operating procedures. Illumina TruSeq Nano is a PCR-based method which, like all PCR-based methods, has limitations compared to PCR-free methods^3–5^. In addition, recent studies have shown that algorithms used to call copy number variation (CNV) from PCR-based library such as EnsembleCNV^6^ can be adapted to identify CNV regions from WGS data^7^. This indicates that PGP-UK WGS data can still be used to call CNV regions with good degree of accuracy.

Sequencing was performed on an Illumina HiSeq X Ten platform with an average depth of 30X. The resulting reads were trimmed using TrimGalore software, mapped to the human reference genome hg19 (GRCh37) using BWA-MEM algorithm (BWA v. 0.7.12^8^). Ambiguously mapped reads (MAPQ <10) and duplicated reads were removed using SAMtools v. 1.2^9^ and Picard v. 1.130 respectively. Genomic variants were called following the Genome Analysis Toolkit software (GATK v. 3.4–46) best practices^10^.

The corresponding FASTQ, BAM and VCF files were deposited in European Nucleotide Archive (ENA) with with study ID PRJEB17529^11^.

### Whole-genome bisulfite sequencing (WGBS)

DNA was extracted from blood samples followed by bisulfite conversion and library preparation using the TruMethyl Whole Genome Kit v2.1. WGBS was performed on an Illumina HiSeq X Ten platform with an average depth of 15X. Generated FASTQ files were processed using GemBS v. 0.11.7 software^12^.

Resulting FASTQ and BAM files were deposited in the ENA with with study ID PRJEB17529^11^.

### RNA Sequencing (RNA-seq)

RNA-seq was performed using 20 ng of RNA isolated from whole blood. All the involved procedures were implemented in accordance with the corresponding manufacturers’ protocols.

Libraries for RNA-seq were prepared with SENSE mRNA-seq Library Prep Kit v2, purified and amplified (18 PCR cycles). After adding adapters and indices, sequencing libraries were further purified using Solid Phase Reversible Immobilisation beads. The output was QC-verified and quantified using Qubit fluorometer. Finally, library QC was performed on Bioanalyzer 2100 and further quantified by qPCR with KAPA library quantification kit and the sequencing was performed on Illumina HiSeq 4000.

RNA-seq FASTQ files are available to download from the ArrayExpress (accession ID E-MTAB-6523^13^) and ENA (project ID PRJEB25139^14^).

### DNA methylation profiling

Genomic DNA (500 ng) extracted from whole blood and saliva was bisulfite converted using the EZ DNA Methylation Kit (Zymo Research) following the recommended incubation conditions for 450 k. Methylation profiling was subsequently performed on 450 k arrays using Illumina iScan Microarray Scanner at UCL Genomics, in accordance with standard operating procedures.

Raw DNA methylation array data (IDAT files) for PGP-UK participants were submitted to the ArrayExpress repository with accession number E-MTAB-5377^15^.

## Data Records

The entire PGP-UK dataset is freely available for download from public repositories with no access restrictions. Links for the particular datasets are provided on the PGP-UK website (www.personalgenomes.org.uk). Accession numbers and dataset identifiers are presented in Table 1. Basic phenotype data, which includes self-reported age, sex, smoking status, etc., alongside with genome and methylome reports, generated by the PGP-UK, can be found on the project’s data web page www.personalgenomes.org.uk/data. Furthermore, all of the data (including associated metadata) are available through the PGP-UK API. The API is compliant with the Open API Specification 3.0 and is documented at www.personalgenomes.org/api.

**Table 1:** PGP-UK data identifiers for the reference panel comprised of 10 PGP-UK participants.

Sample ID	EBI ID	Tissue	WGS	WGBS	450 k	RNA-seq
			ENA PRJEB17529	ENA PRJEB17529	Array Express E-MTAB-5377	ENA PRJEB25139
uk35C650	SAMEA4545245	blood	ERX1796409	ERX2408504	101130760050_R04C02	ERX2373318
		saliva			101130760049_R03C01	
uk2E2AAE	SAMEA4545246	blood	ERX1796410	ERX2408505	101130760050_R05C02	ERX2373321
		saliva			101130760050_R03C01	
uk2DF242	SAMEA4545247	blood	ERX1796411	ERX2408506	101130760049_R06C02	ERX2373317
		saliva			101130760049_R03C02	
uk740176	SAMEA4545248	blood	ERX1796412	ERX2408507	101130760050_R06C02	ERX2373324
		saliva			101130760050_R06C01	
uk33D02F	SAMEA4545249	blood	ERX1796413	ERX2408508	101130760049_R05C02	ERX2373316
		saliva			101130760049_R04C02	
uk0C72FF	SAMEA4545250	blood	ERX1796414	ERX2408509	101130760049_R06C01	ERX2373322
		saliva			101130760050_R01C01	
uk1097F9	SAMEA4545251	blood	ERX1796415	ERX2408510	101130760050_R02C01	ERX2373320
		saliva			101130760050_R01C02	
uk174659	SAMEA4545252	blood	ERX1796416	ERX2408511	101130760050_R05C01	ERX2373325
		saliva			101130760049_R05C01	
uk85AA3B	SAMEA4545253	blood	ERX1796417	ERX2408512	101130760049_R02C02	ERX2373323
		saliva			101130760049_R01C01	
uk481F67	SAMEA4545254	blood	ERX1796418	ERX2408513	101130760049_R02C01	ERX2373319
		saliva			101130760050_R02C02	

For each of them WGS, WGBS and RNA-seq data were obtained from blood samples, methylation profiles were obtained using 450 k from both blood and saliva samples. The table contains ENA accession numbers for WGS, WGBS and RNA-seq, for 450 k data it shows Sentrix IDs and positions, separated by the underscores.

Whole genome sequencing and whole genome bisulfite sequencing data are freely available from the ENA under the project ID PRJEB17529^11^. RNA-seq data is deposited in ArrayExpress under the accession number E-MTAB-6523^13^ and in ENA PRJEB25139^14^. DNA methylation array data for PGP-UK participants is stored in ArrayExpress under the accession number E-MTAB-5377^15^.

The PGP-UK pilot dataset described in^2^ resulted in the PGP-UK multi-omics reference panel described here. The datasets are available from the above-mentioned repositories and from the Seven Bridges Cancer Genomics cloud (docs.cancergenomicscloud.org/docs/personal-genome-project-uk-pgp-uk-pilot-dataset), which offers various tools and workflows for genomic and epigenomic data analysis.

The PGP-UK multi-omics reference panel is also available in the Lifebit cloud through their Open Data project (opendata.lifebit.ai/table/pgp) along with interactive analyses (ancestry, phenotypic traits, genetic variance) and custom pipelines provided by Lifebit’s cloud-computing platform Deploit (deploit.lifebit.ai). As a part of our collaboration with Lifebit our data have also been uploaded to a public Amazon Web Services (AWS) Simple Storage Service (S3) Bucket. This S3 Bucket is available at https://s3.console.aws.amazon.com/s3/buckets/pgp-lifebit (publically accessible with an AWS account) and can be used independently of the Lifebit platform within AWS or any other cloud platform using AWS S3 APIs.

To provide maximum access, PGP-UK data can in principle be hosted in any cloud complying with ‘best practices’ as well as adequate legal and ethical governance^16^. To this end, we have initiated discussions to also host our data under the Early Adopter Programme of the European Open Science Cloud (https://www.eosc-portal.eu/) and with Open Humans (https://www.openhumans.org/) which opened their project to global members in March 2019.

## Technical Validation

In this section, we describe the outcomes of the PGP-UK data quality control checks and validation for the pilot cohort. In a first instance, we describe the QC framework and discuss outputs for each types of data collected. Then, we provide details of multi-omics data matching validation procedures based on cross-comparison of variants between different data types for each individual.

### Data quality control

#### WGS data QC

Quality control of the reported WGS data was performed using FastQC v. 0.11.2 (https://www.bioinformatics.babraham.ac.uk/projects/fastqc/) and Picard v. 1.130 (https://github.com/broadinstitute/picard) tools. QC reports were generated using MultiQC v. 1.5 software^17^.

**Fig. 2:**
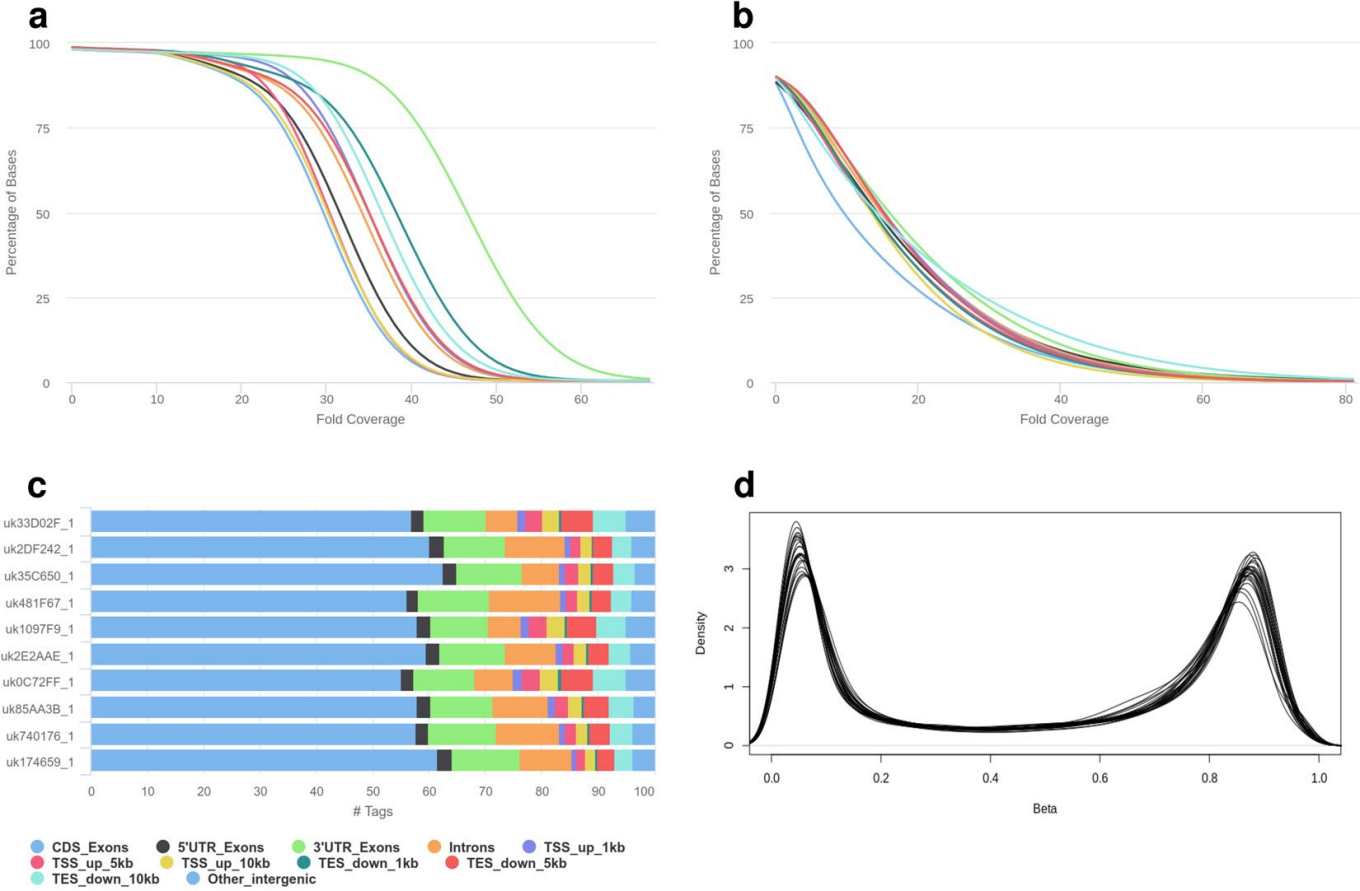
PGP-UK QC images for WGS, WGBS, RNA-seq and 450 k methylation data. (**a**) WGS coverage depth plot. (**b**) WGBS coverage depth plot. (**c**) RNA-seq reads distribution over the different genome features. (**d**) Density plot for Illumina 450 k methylation profiles.

The WGS data average median coverage is above 35X (varies between 30X and 47X across samples) with more than 73% of the bases covered reaching 30X or more (varies between 54% and 95% across samples), see Fig. 2(a). A summary of the WGS QC analysis is presented in Table 2.

**Table 2:** Quality control metrics summary of the WGS data derived from blood samples of 10 PGP-UK participants.

Sample ID	Median Coverage	Bases ≥30X	Duplicated Reads, %		GC Content, %	
			Read 1	Read 2	Read 1	Read 2
uk35C650	32.0X	64%	8.0%	6.3%	40%	41%
uk2E2AAE	47.0X	95%	18.3%	18.4%	41%	41%
uk2DF242	35.0X	75%	10.2%	13.6%	41%	41%
uk740176	35.0X	80%	8.3%	9.6%	40%	41%
uk33D02F	31.0X	58%	11.2%	12.1%	41%	41%
uk0C72FF	31.0X	57%	3.7%	8.1%	41%	41%
uk1097F9	39.0X	85%	4.5%	12.7%	40%	41%
uk174659	35.0X	78%	8.5%	15.1%	41%	41%
uk85AA3B	37.0X	85%	6.1%	3.2%	41%	41%
uk481F67	30.0X	54%	8.6%	7.0%	41%	41%

The table contains median coverage depth, percentage of bases covered with at least 30X depth, as well as duplicated reads and GC contents percentages for both forward and reverse reads for each sample.

#### WGBS data QC

GemBS v. 3.2.1, FastQC v. 0.11.7 and Picard v. 2.18.23 tools were used in quality control of the PGP-UK WGBS and data QC reports were generated using MultiQC v. 1.5 software^17^.

WGBS average median coverage is above 14X (ranging from 10X to 16X across samples) with more than 19% of bases covered reaching 30X or deeper (varies between 15% and 25% across samples), see Fig. 2(b). Summary of the WGBS QC analysis is presented in Table 3.

**Table 3:** Quality control metrics summary of the WGBS data derived from blood samples of 10 PGP-UK participants.

Sample ID	Median Coverage	Bases ≥30X	Duplicated Reads, %		GC Content, %	
			Read 1	Read 2	Read 1	Read 2
uk35C650	10.0X	15%	27.3%	13.3%	26%	29%
uk2E2AAE	15.0X	20%	39.4%	20.3%	24%	27%
uk2DF242	16.0X	23%	28.0%	12.4%	24%	27%
uk740176	15.0X	20%	25.8%	12.6%	25%	27%
uk33D02F	16.0X	20%	26.3%	13.1%	24%	27%
uk0C72FF	14.0X	18%	26.8%	11.4%	25%	28%
uk1097F9	14.0X	15%	26.0%	10.8%	24%	27%
uk174659	14.0X	17%	27.1%	15.5%	24%	27%
uk85AA3B	16.0X	19%	28.3%	14.9%	24%	27%
uk481F67	15.0X	25%	31.6%	17.4%	26%	29%

The table contains median coverage depth, percentage of bases covered with at least 30X depth, as well as duplicated reads and GC contents percentages for both forward and reverse reads for each sample.

#### RNA-seq data QC

All of the RNA-seq samples were processed with a modified version of the nextflow^18^ nf-core RNA-seq pipeline (https://github.com/UCL-BLIC/rnaseq). Specifically, reads were trimmed with TrimGalore v. 0.4.1, aligned against hg19 with STAR v. 2.5.2a^19^ and duplicated reads were identified and removed with Picard v. 2.18.9 tools. QC reports were generated using MultiQC v. 1.5^17^ as part of the same pipeline. Reads were further split and trimmed using GATK4.

The mean RNA integrity number (RIN) value of the RNA used for sequencing was 8.55 (ranging between 7.1 and 9.3). Figure 2(c) demonstrates the distribution of mapped reads over various genomic features. A summary of the RNA-seq QC analysis is presented in Table 4.

**Table 4:** Quality control metrics summary of the RNA-seq data derived from blood samples of 10 PGP-UK participants.

Sample ID	RIN	Uniquely Aligned, %	Duplicated Reads, %		GC Content, %	
			Read 1	Read 2	Read 1	Read 2
uk35C650	8.8	88.8%	83.2%	80.6%	53%	56%
uk2E2AAE	9.1	89.3%	85.9%	82.3%	53%	56%
uk2DF242	9.2	90.0%	86.3%	81.9%	53%	56%
uk740176	8.5	90.0%	84.8%	80.6%	53%	56%
uk33D02F	8.3	87.0%	85.5%	82.6%	53%	56%
uk0C72FF	7.9	86.7%	85.0%	82.5%	53%	56%
uk1097F9	8.7	86.1%	86.5%	82.6%	54%	57%
uk174659	9.3	90.4%	84.4%	81.3%	53%	56%
uk85AA3B	8.6	89.0%	84.9%	81.2%	53%	56%
uk481F67	7.1	90.4%	87.3%	83.7%	52%	55%

The table contains RIN value, percentages of uniquely aligned bases, as well as duplicated reads and GC contents percentages for both forward and reverse reads for each sample.

#### 450 k methylation data QC

450 k DNA methylation profiles were generated from whole blood and saliva for each of the ten participants in the PGP-UK multi-omics reference panel. For quality control of these data, we used R v. 3.5.2 with minfi v. 1.28.3 and ewastools v. 1.4 libraries^20,21^.

We performed quality checks based on 17 metrics assessed at control probes as described in the Illumina’s BeadArray Controls Reporter. All 17 metrics derived from the array control probes’ data are within the manufacturer’s recommended thresholds. In addition, we analysed detection *p*-values and bead count information, which is available for 100% and 99.92% of CpGs respectively. 99.96% of the detection *p*-values are below the threshold of 0.01. Average CpG bead count number across all samples is 14, and 100% of the available bead count numbers ≥3. A summary of this analysis is presented in Table 5. Figure 2(d) shows the overlay of the *β*-value density distributions for all samples.

**Table 5:** Quality control metrics summary of the Illumina 450 k data derived from blood and saliva samples of 10 PGP-UK participants.

Sample ID	Tissue	Detection *p*-values		Bead Count	
		Available, %	*p* < 0.01, %	Available, %	*n* ≥ 3, %
uk35C650	blood	100%	99.98476%	99.91370%	100%
	saliva	100%	99.97899%	99.93710%	100%
uk2E2AAE	blood	100%	99.92297%	99.92503%	100%
	saliva	100%	99.93491%	99.93670%	100%
uk2DF242	blood	100%	99.96478%	99.90896%	100%
	saliva	100%	99.97178%	99.92110%	100%
uk740176	blood	100%	99.91638%	99.90361%	100%
	saliva	100%	99.91638%	99.91800%	100%
uk33D02F	blood	100%	99.92791%	99.91761%	100%
	saliva	100%	99.92771%	99.93120%	100%
uk0C72FF	blood	100%	99.97467%	99.90484%	100%
	saliva	100%	99.98558%	99.88910%	100%
uk1097F9	blood	100%	99.98929%	99.92460%	100%
	saliva	100%	99.98744%	99.92150%	100%
uk174659	blood	100%	99.97714%	99.94151%	100%
	saliva	100%	99.97899%	99.92190%	100%
uk85AA3B	blood	100%	99.93327%	99.89351%	100%
	saliva	100%	99.94089%	99.91670%	100%
uk481F67	blood	100%	99.98126%	99.91514%	100%
	saliva	100%	99.98105%	99.92130%	100%

The table contains percentages of available detection *p*-values and bead count numbers together with percentages of *p* < 0.01 and bead count numbers 3 and above for each sample.

### Multi-omics data matching

**Fig. 3:**
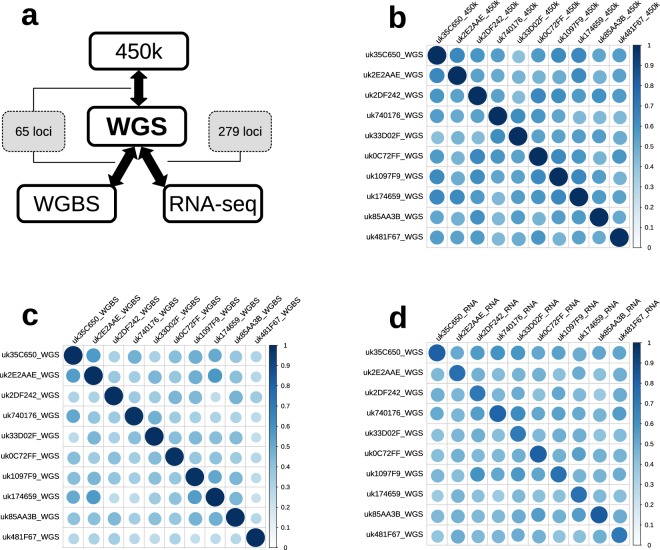
Multi-Omics Data Matching. (**a**) Multi-Omics Data Matching Schema. 65 loci were used in matching WGS with methylation and WGBS data, 279 loci were used in matching WGS with RNA-seq data. (**b**) Correlation plot displaying matching results for WGS vs. 450 k datasets. (**c**) Correlation plot displaying matching results for WGS vs. WGBS datasets. (**d**) Correlation plot displaying matching results for WGS vs. RNA-seq datasets. On correlation plots (**b**–**d**) scale is represented by the combination of ball size and colour (from white to dark blue) and goes from 0 (0% match) to 1 (perfect 100% match).

In order to ensure data integrity and exclude the possibility of sample mix-up between study participants, we validated our sample assignments, by matching the available 450 k, WGBS and RNA-seq data against WGS. First, we matched the 450 k against WGS data for each participant using 65 single nucleotide polymorphisms (SNP) control probes from Illumina 450 k array. Second, we matched the WGBS-derived genotypes for the same 65 SNP loci with the WGS data. Third, we compared genotypes derived from RNA-seq and WGS data based on the set of loci from protein coding regions. The schema of the multi-omics data matching is given in Fig. 3(a) and further details are provided below.

We used *β*-values recorded at the 65 450 k SNP control probes to distinguish between heterozygous and homozygous alleles in the 450 k dataset. These SNPs are by design highly variable and can therefore provide a unique genetic signature that can be used to differentiate between each study participant. Note that 64 out of these 65 SNPs are outside protein-coding regions and, hence, not available for the RNA-seq data. We identified 279 SNP loci present in at least 4 WGS samples which were also highly expressed across RNA-seq samples (in the top 100 most expressed genes) yielding a suitable validation set for the WGS vs. RNA-seq comparison.

To match the different datasets, we extracted the locations of the loci used for validation (65 loci for the WGS vs. 450 k and WGBS vs. 450 k comparisons, and 279 loci for WGS vs. RNA-seq) and used the HaplotypeCaller and GenotypeGVCFs tools from (GATK v. 3.8.0) on the corresponding BAM files to force the call of genotypes in these locations. Percentage of matching genotypes were then obtained across samples and datasets to confirm sample identity as presented on Fig. 3 and Table 6.

**Table 6:** Summary of data cross-validation between 450 k, WGBS and RNA-seq against WGS.

Sample ID	WGS vs. 450 k			WGS vs. WGBS			WGS vs. RNA-seq		
	Loci, n	Loci, %	matched, %	Loci, n	Loci, %	matched, %	Loci, n	Loci, %	matched, %
uk35C650	65	100	100	52	80	100	161	57.71	81.99
uk2E2AAE	65	100	100	51	78.46	100	172	61.65	75.58
uk2DF242	65	100	100	58	89.23	100	183	65.59	70.49
uk740176	65	100	100	61	93.85	98.36	152	54.48	80.26
uk33D02F	65	100	100	53	81.54	100	188	67.38	69.68
uk0C72FF	65	100	100	57	87.69	98.25	159	56.99	81.13
uk1097F9	65	100	100	54	83.08	98.15	190	68.10	73.68
uk174659	65	100	100	52	80	100	197	70.61	74.62
uk85AA3B	65	100	100	60	92.30	100	167	59.86	83.23
uk481F67	65	100	100	53	81.54	100	169	60.57	71.01

Columns Loci, n and Loci, % contain respective numbers and percentages of loci used for matching (out of 65 loci for WGS and WGBS vs. 450 k and out of 279 loci for WGS vs. RNA-seq).

#### WGS vs. 450 k

In order to obtain genotype information from 450 k data, we extracted *β*-values for the 65 SNP control probes for each of the 10 PGP-UK participants. As expected, these *β*-values clustered into three separate peaks around 0.5 (which corresponds to heterozygous genotypes), 0 and 1 (which correspond to homozygous genotypes). We checked and confirmed that reported *β*-values for all SNP control probes which were derived from the whole blood and corresponding saliva 450 k datasets were a 100% match. In other words, we established that the zygosity of each probe was the same across both DNA samples for any given participant.

We then extracted the genotypes for those 65 SNPs from WGS and matched them with to the corresponding zygosity in the 450 k data. This resulted in perfect 100% match for corresponding samples, i.e. samples from the same participant, see Fig. 3(b) and Table 6.

#### WGS vs. WGBS

This comparison was performed by matching WGS- and WGBS-derived genotypes for 65 Illumina 450 k array SNP control probes. The mean agreement between matched samples was 99.45%, which corresponds to a total of 3 loci mismatch observed in 3 out of ten participants (i.e. a single mismatch for each of those three participants). Altogether, 100% and 84.77% of 65 SNPs had coverage in the WGS and WGBS data respectively, which allowed us to make our comparison based on 51–61 common loci per participant, see Fig. 3(c) and Table 6.

#### WGS vs. RNA-seq

To match RNA-seq with WGS samples, we used a set of common loci in highly expressed genes as described above. Available genotypes for these loci were extracted from the RNA-seq and WGS samples and cross-validated. In total, 92.65% and 80.93% of these 279 loci had coverage in the RNA-seq and WGS data respectively, which allowed us to make our comparison based on 152–197 loci per participant. On average, corresponding WGS and RNA-seq data are in agreement for 76.17% of genotype calls (range 69.68–83.23%), see Fig. 3(d) and Table 6.

Results of matching 450 k, RNA-seq and WGBS data with WGS are presented in Table 6. The correlation plots presented on Fig. 3, demonstrate a substantially higher level of correspondence between samples from the same individual compared to those from different people when comparing WGS vs. 450 k (Fig. 3(b)), WGS vs. WGBS (Fig. 3(c)) and WGS vs. RNA-seq (Fig. 3(d)).

## Usage Notes

Here we describe two key outputs generated for each PGP-UK participant, the Genome and Methylome Reports. These reports are freely available to download on PGP-UK website, see https://www.personalgenomes.org.uk/data/.

Genome Reports leverage the information from variant call files (VCFs) and provide an overview of the potential influence of genetic variants on several genetic traits, as well as ancestry information. Potentially beneficial or harmful traits for each participant were identified using public data from SNPedia^22^, gnomAD v2.0.2^23^, GetEvidence^24^ and ClinVar^25^. Plots to visualise the ancestry of each participant were created by applying principal component analysis (as implemented in Plink v1.9^26^) on a genotype matrix resulting from merging the participant genotypes with those from 2504 unrelated samples from 26 worldwide populations available from the 1000 Genomes Project^27^. Population membership proportions were obtained using the Admixture v1.3.0 software^28^ on above-mentioned genotype matrix.

Methylome reports contain epigenetic age and smoking status prediction for PGP-UK participants based on their methylome as assessed by 450 k array experiments. Raw data were processed, quality controlled and analysed using ChAMP^29,30^ and minfi^20^ pipelines for R. Epigenetic age calculation was based on the multi-tissue Horvath clock^31^, which predicts age using a linear combination of the methylation levels from a reference panel of 353 CpGs. Smoking status was predicted by calculating smoking scores as linear combinations of the methylation levels at 183 CpGs and then comparing them to a particular threshold as described in^32^. More details on the PGP-UK Genome and Methylome reports are described in^2^.

## Outlook

Because of its open access status, the PGP-UK multi-omics reference panel described here has the potential to become the reference panel of choice for the implementation of the FAIR (Findable, Accessible, Interoperable, Reusable) principles^33^ for data sharing and the integration of new data standards and formats. While we shall aim to increase the size of the panel in the longer term, the immediate aim is to add more data. Towards this, we have already generated methylation count files (MCFs) which are the epigenetic equivalent to variant count files (VCFs). These pre-processed data files are very popular with users for downstream analyses. While agreed standards and procedures are in place for generating and depositing VCFs into public databases, PGP-UK is at the forefront of helping to establish these for MCFs in collaboration with EBI (https://www.ebi.ac.uk) and ELIXIR (https://elixir-europe.org/). In addition, PGP-UK is spearheading efforts to add Phenopackets to our reference panel. PhenoPackets are represented as phenotype exchange files (PXFs), a novel open standard for sharing disease and phenotype information (http://phenopackets.org). For disease and health information in general, we are currently exploring with Patients Know Best (https://www.patientsknowbest.com/) how to link our reference panel to the corresponding NHS health records. All these activities are conducted in compliance and collaboration with EU standards for precision medicine, EU-STANDS4PM (https://www.eu-stands4pm.eu/).

## Data Availability

All the PGP-UK data pre-processing, QC and analyses were performed with publicly available software packages, using versions and parameters described in the paper.
